# Supplementary material for: Intercellular communication atlas reveals Oprm1 as a neuroprotective factor for retinal ganglion cells
Source: Res Sq. 2023 Aug 17:rs.3.rs-3193738. Preprint. [Version 1] doi: 10.21203/rs.3.rs-3193738/v1 (PMC10462234; doi:10.21203/rs.3.rs-3193738/v1)
Supplement: Supplement 1 [file NIHPPrs3193738v1-supplement-1.pdf]

Supplementary Figures

Supplementary Fig. 1: Retinal cells in response to ONC.
Response in astrocytes **a**, microglia **b,** and glycinergic amacrine cells **c**, as some examples, after the ONC injury on RGCs. Left panels: DEG heatmaps show dynamical patterns of DEGs at different time points after ONC. Middle panels: Gene ontology analysis reveals representative biological processes enriched by the DEGs in each pattern shown in the heatmaps. Right panel: Density plots of the absolute fold-change (log2FC) values of genes expressed (detection rate > 0.1). Ligand and receptor genes (L/R) shown with yellow color are grouped together, while the other genes are shown with light purple for comparison.

Supplementary Fig. 2: Responsive interactions from other cells to RGCs.
a, Correlation of gene expression between RGCs captured and sequenced in whole retina data in this paper and the RGCs in Tran. et al. **b,** Additional examples of cell-cell feedback loops. The ligand from the sender cell interacts with the receptor on the receiver cell, triggering gene transcription in receiver cells, in which some ligand genes are transcribed and sent back to the original sender cells. **c,** The number of ligand-receptor interactions from other retinal cells to RGCs. Interactions identified in multiple and unique cell types are colored dark blue and orange, respectively. **d,** The specific ligand-receptor interactions identified in unique retinal cell type to RGCs, which are shown in orange color columns in the panel c. Genes in the bottom half of the circle are the ligands secreted from other retinal cells, and the genes in the top half of the circle are the receptors in RGCs. The colors of the connecting edges represent the receiver cell types. **e,** Heatmap reveals the ONC-induced temporal patterns of interactions from astrocytes to RGCs across time points (the fold change (FC) of the interaction scores between any two time points > 1.2). Four dynamical patterns were identified. **f,** Gene Ontology analysis (GO) reveals representative biological processes of variable ligand-receptor interactions in patterns identified in panel e. The enrichment was calculated with all expressed genes in either astrocyte or RGC in any time point as the background (detection rate > 0.1).

Supplementary Fig. 3: Properties of high-survival vs low-survival RGC subclasses.
a, Number of expressed ligand and receptor genes in RGC subclasses (detection rate > 0.1 in any time point). **b,** Additional examples for calculating protective interactions in astrocytes (left graph), microglia (middle graph) and glycinergic amacrine cells (right graph). For each ligand-receptor pair, the two calculated mean values of interaction scores, one (y-axis) for the high- and the other (x-axis) for the low-survival RGC subclasses were plotted for comparison. Blue dots are the interactions that are stronger in high- than in low-survival RGC subclasses. Gray dots are the interactions with similar interaction scores between the two categories.

Supplementary Fig. 4: Features of the protective interactions.
a, Average expression level of ligand genes in RGC subclasses. **b,** Summary of the preset and induced protective interactions. X-axis is the difference between interaction scores in high- and low-survival subclasses before injury, and Y-axis is the interaction score difference after injury. Interactions from GABAergic amacrine cells to RGCs are shown in upper graph, and the Müller glia to RGCs interactions are shown in lower graph.

Supplementary Fig. 5: Neuroprotective effect of Oprm1 on RGC survival.
a, Representative confocal images of retina whole mounts of RBPMS staining 5 days (upper row) or 14 days (lower row) post-ONC. **b,** Representative confocal images of retina whole

mounts of RBPMS staining 7-days following NMDA damage. **c,** Representative confocal scans of petal-shape retina whole mounts showing the OPN4 staining of ipRGC, four days after NMDA damage, compared with co-treatment with NMDA and naloxone injection. White dash-line circles label the ipRGCs (OPN4 in red fluorescence). **d,** Representative confocal images of retina whole mounts showing ipRGC numbers five days post-ONC. The red channel represents OPN4 staining, and the green fluorescence shows vGlut2-Sun1GFP as pan-RGC marker. White dashed line circles mark the ipRGCs. **e,** Numbers of ipRGC cell survived (OPN4+), as the percentages relative to the sham group. Data are presented as mean ± SEM. ONC group, n=5; ONC+ Nx group, n=6. One-Way ANOVA, multiple comparison, *p < 0.05. **f,** Representative confocal scans of petal-shape retina whole mounts showing ipRGC cell survived 5-days post-ONC, and ONC combined with naloxone injection condition. White dashed line circles label the ipRGCs (OPN4 in red fluorescence).

Supplementary Fig. 6: snRNA-seq analysis on sorted RGC nuclei.
a, Retinal cells obtained from the samples. The samples were enriched for RGCs with anti-GFP MACS for vGlut2-Sun1GFP+ pan-RGCs. **b,** Expression level of representative known marker genes in retinal cell types. **c,** Expression pattens of ectopic human Oprm1 (based on AAV2 WPRE detection) in retinal cells. The ectopic Oprm1 is mainly detected in RGCs.

Supplementary Fig. 7: AAV-mediated *Oprm1* gene transduction in eIOP glaucoma model.
a, Representative confocal images of retina whole mounts showing the expression of mCherry in Oprm1 + eIOP group, which was transduced with AAV2-FLEX-mCherry-Oprm1 on vGlut2-Cre;LSL-Sun1GFP mice. The red fluorescent staining is mCherry, green fluorescence is Sun1GFP in pan-RGCs, and the blue channel represents RBPMS staining. **b,** Representative confocal fluorescent images of retina whole mounts showing the expression of mCherry in the Oprm1 + eIOP group, which was transduced with AAV2-FLEX-mCherry-Oprm1, on vGlut2-Cre;LSL-Sun1GFP mice. The red fluorescent staining is mCherry, green fluorescence is Sun1GFP in pan-RGCs, and the blue channel represents Oprm1 staining.

Suppl Fig. 1

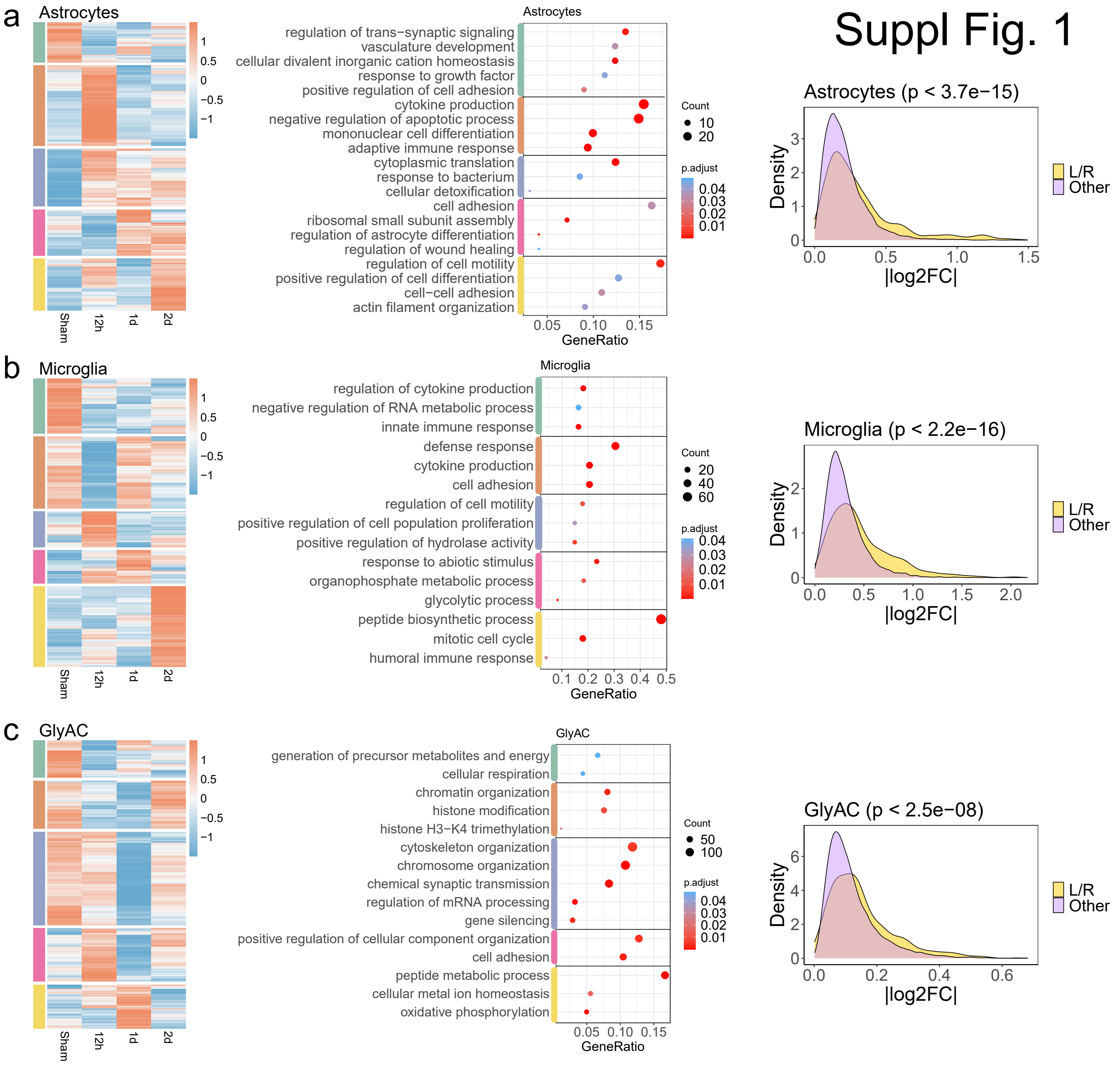
a
Astrocytes
1
0.5
0
−0.5
−1
Sham
12h
1d
2d
Astrocytes
regulation of trans−synaptic signaling
vasculature development
cellular divalent inorganic cation homeostasis
response to growth factor
positive regulation of cell adhesion
cytokine production
negative regulation of apoptotic process
mononuclear cell differentiation
adaptive immune response
cytoplasmic translation
response to bacterium
cellular detoxification
cell adhesion
ribosomal small subunit assembly
regulation of astrocyte differentiation
regulation of wound healing
regulation of cell motility
positive regulation of cell differentiation
cell−cell adhesion
actin filament organization
0.05
0.10
0.15
GeneRatio
Count
10
20
p.adjust
0.04
0.03
0.02
0.01
Astrocytes (p < 3.7e−15)
Density
|log2FC|
L/R
Other
b
Microglia
Sham
12h
1d
2d
Microglia
regulation of cytokine production
negative regulation of RNA metabolic process
innate immune response
defense response
cytokine production
cell adhesion
regulation of cell motility
positive regulation of cell population proliferation
positive regulation of hydrolase activity
response to abiotic stimulus
organophosphate metabolic process
glycolytic process
peptide biosynthetic process
mitotic cell cycle
humoral immune response
0.1
0.2
0.3
0.4
0.5
GeneRatio
Count
20
40
60
Microglia (p < 2.2e−16)
c
GlyAC
Sham
12h
1d
2d
GlyAC
generation of precursor metabolites and energy
cellular respiration
chromatin organization
histone modification
histone H3−K4 trimethylation
cytoskeleton organization
chromosome organization
chemical synaptic transmission
regulation of mRNA processing
gene silencing
positive regulation of cellular component organization
cell adhesion
peptide metabolic process
cellular metal ion homeostasis
oxidative phosphorylation
Count
50
100
GlyAC (p < 2.5e−08)

Suppl Fig. 2

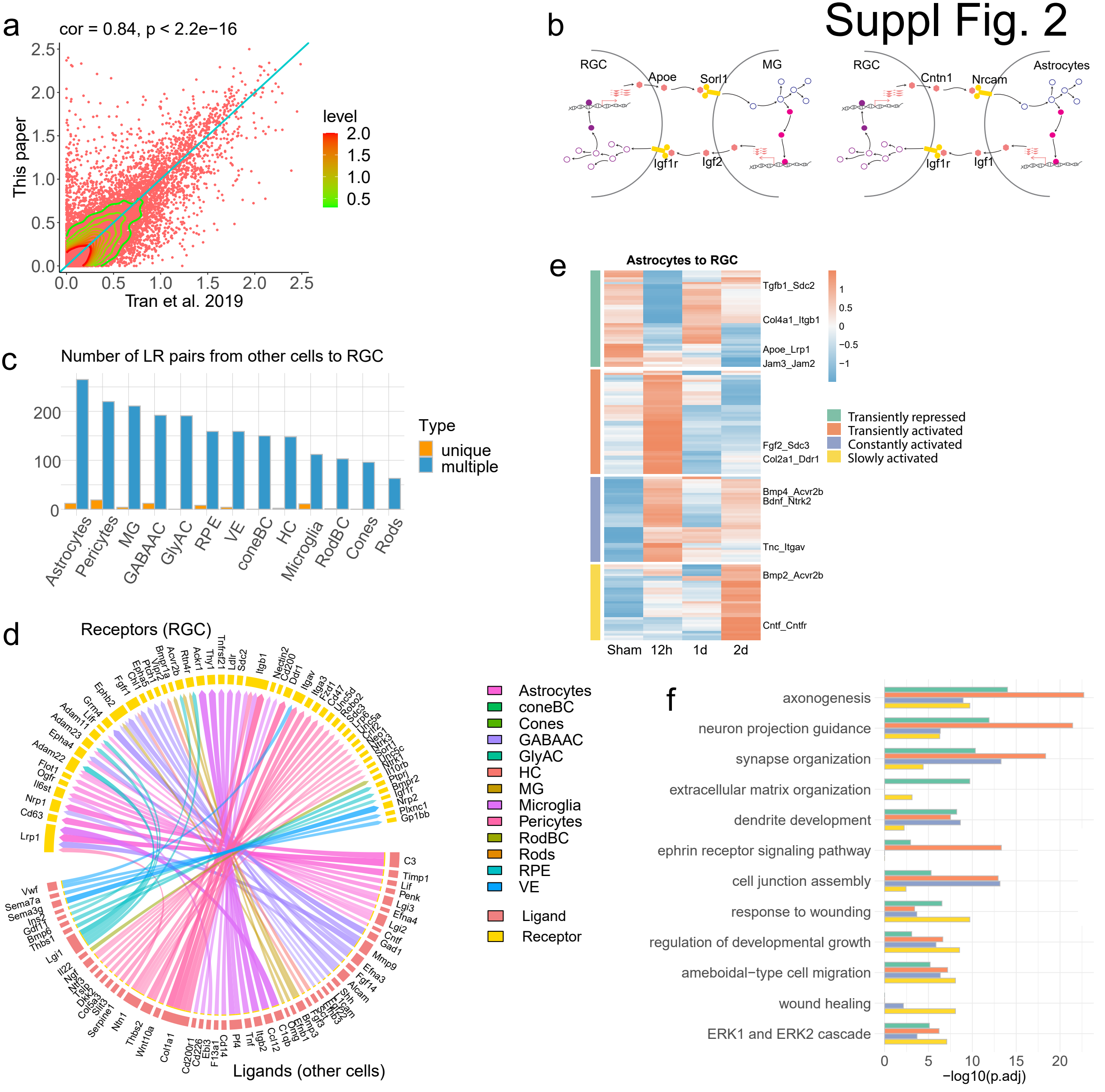
a
cor = 0.84, p < 2.2e−16
This paper
Tran et al. 2019
level
2.0
1.5
1.0
0.5
b
RGC
Apoe
Sorl1
MG
Igf1r
Igf2
RGC
Cntn1
Nrcam
Astrocytes
Igf1r
Igf1
c
Number of LR pairs from other cells to RGC
Type
unique
multiple
Astrocytes
Pericytes
MG
GABAAC
GlyAC
RPE
VE
coneBC
HC
Microglia
RodBC
Cones
Rods
d
Receptors (RGC)
Ligands (other cells)
Astrocytes
coneBC
Cones
GABAAC
GlyAC
HC
MG
Microglia
Pericytes
RodBC
Rods
RPE
VE
Ligand
Receptor
e
Astrocytes to RGC
Tgfb1_Sdc2
Col4a1_Itgb1
Apoe_Lrp1
Jam3_Jam2
Fgf2_Sdc3
Col2a1_Ddr1
Bmp4_Acvr2b
Bdnf_Ntrk2
Tnc_Itgav
Bmp2_Acvr2b
Cntf_Cntfr
Sham
12h
1d
2d
Transiently repressed
Transiently activated
Constantly activated
Slowly activated
f
axonogenesis
neuron projection guidance
synapse organization
extracellular matrix organization
dendrite development
ephrin receptor signaling pathway
cell junction assembly
response to wounding
regulation of developmental growth
ameboidal−type cell migration
wound healing
ERK1 and ERK2 cascade
−log10(p.adj)

Suppl Fig. 3

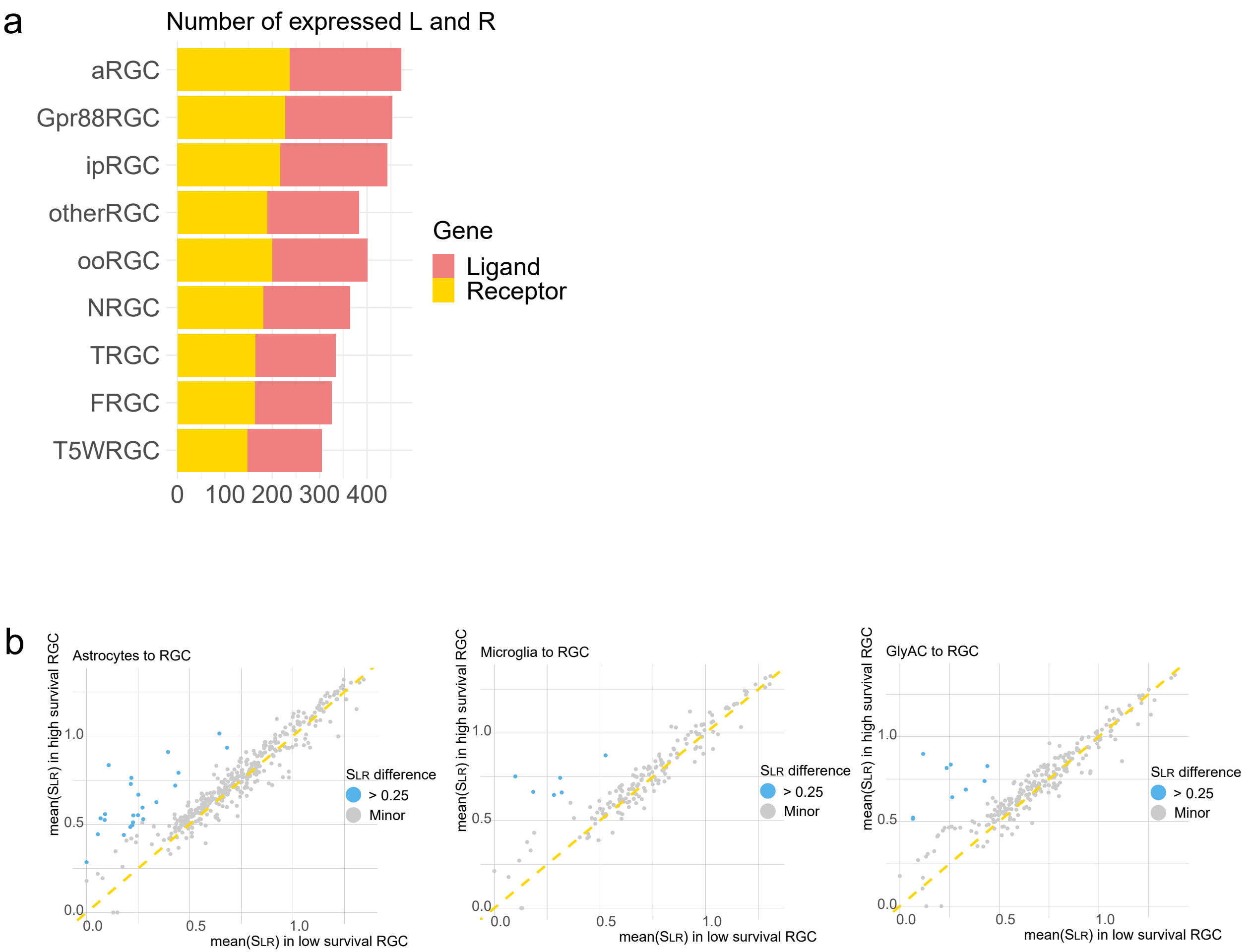
a
Number of expressed L and R
aRGC
Gpr88RGC
ipRGC
otherRGC
ooRGC
NRGC
TRGC
FRGC
T5WRGC
0
100
200
300
400
Gene
Ligand
Receptor
b
Astrocytes to RGC
Microglia to RGC
GlyAC to RGC
mean(SLR) in high survival RGC
mean(SLR) in low survival RGC
0.0
0.5
1.0
SLR difference
> 0.25
Minor

Suppl Fig. 4

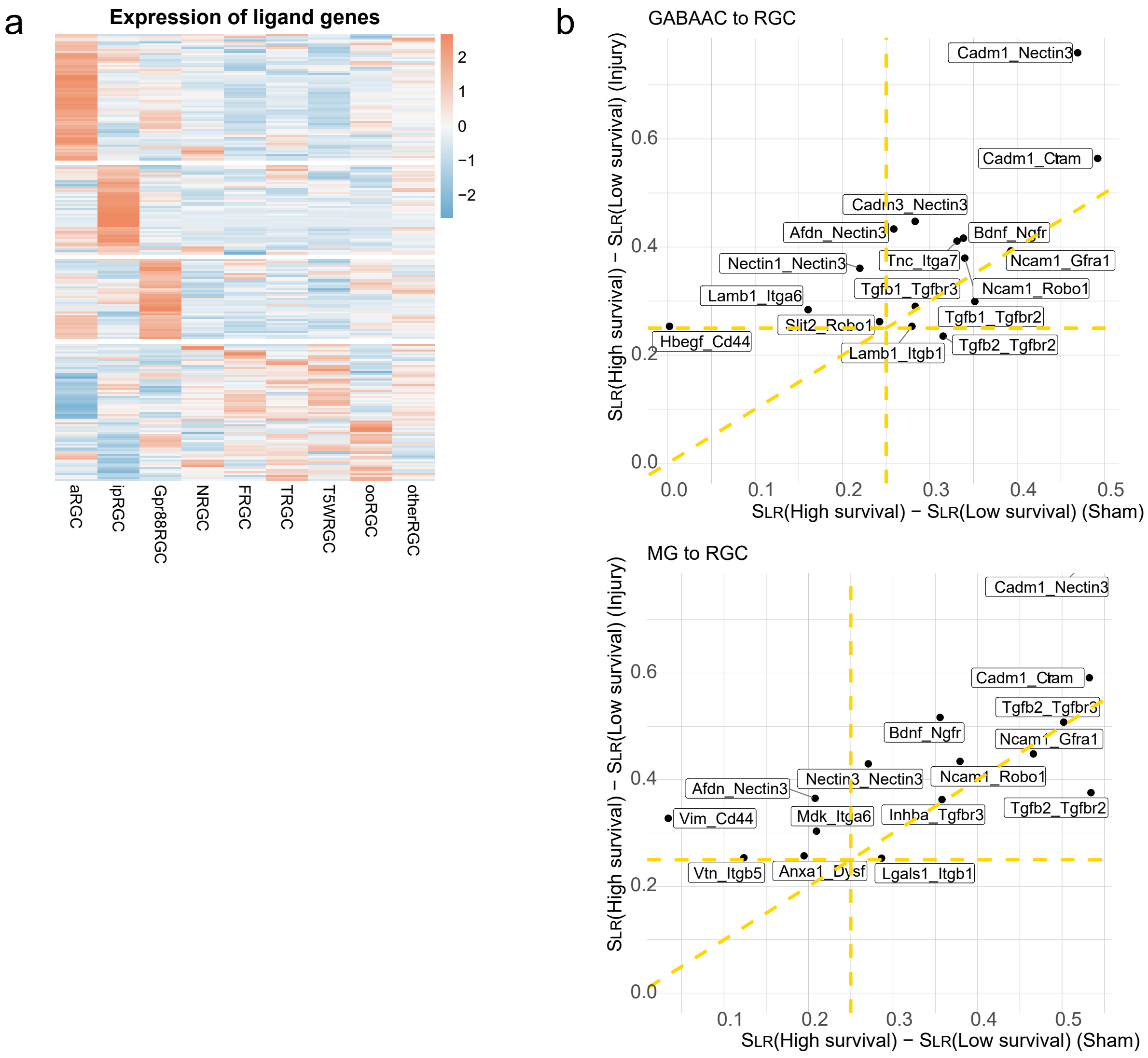

a
Expression of ligand genes
2
1
0
−1
−2
aRGC
ipRGC
Gpr88RGC
NRGC
FRGC
TRGC
T5WRGC
ooRGC
otherRGC
b
GABAAC to RGC
Cadm1_Nectin3
Cadm1_Cram
Cadm3_Nectin3
Afdn_Nectin3
Bdnf_Ngfr
Nectin1_Nectin3
Tnc_Itga7
Ncam1_Gfra1
Lamb1_Itga6
Tgfb1_Tgfbr3
Ncam1_Robo1
Tgfb1_Tgfbr2
Slit2_Robo1
Hbegf_Cd44
Tgfb2_Tgfbr2
Lamb1_Itgb1
0.0
0.2
0.4
0.6
0.0
0.1
0.2
0.3
0.4
0.5
SLR(High survival) − SLR(Low survival) (Sham)
SLR(High survival) − SLR(Low survival) (Injury)
MG to RGC
Cadm1_Nectin3
Cadm1_Cram
Tgfb2_Tgfbr3
Bdnf_Ngfr
Ncam1_Gfra1
Nectin3_Nectin3
Ncam1_Robo1
Afdn_Nectin3
Vim_Cd44
Mdk_Itga6
Inhba_Tgfbr3
Tgfb2_Tgfbr2
Vtn_Itgb5
Anxa1_Dysf
Lgals1_Itgb1
0.0
0.2
0.4
0.6
0.1
0.2
0.3
0.4
0.5
SLR(High survival) − SLR(Low survival) (Sham)
SLR(High survival) − SLR(Low survival) (Injury)

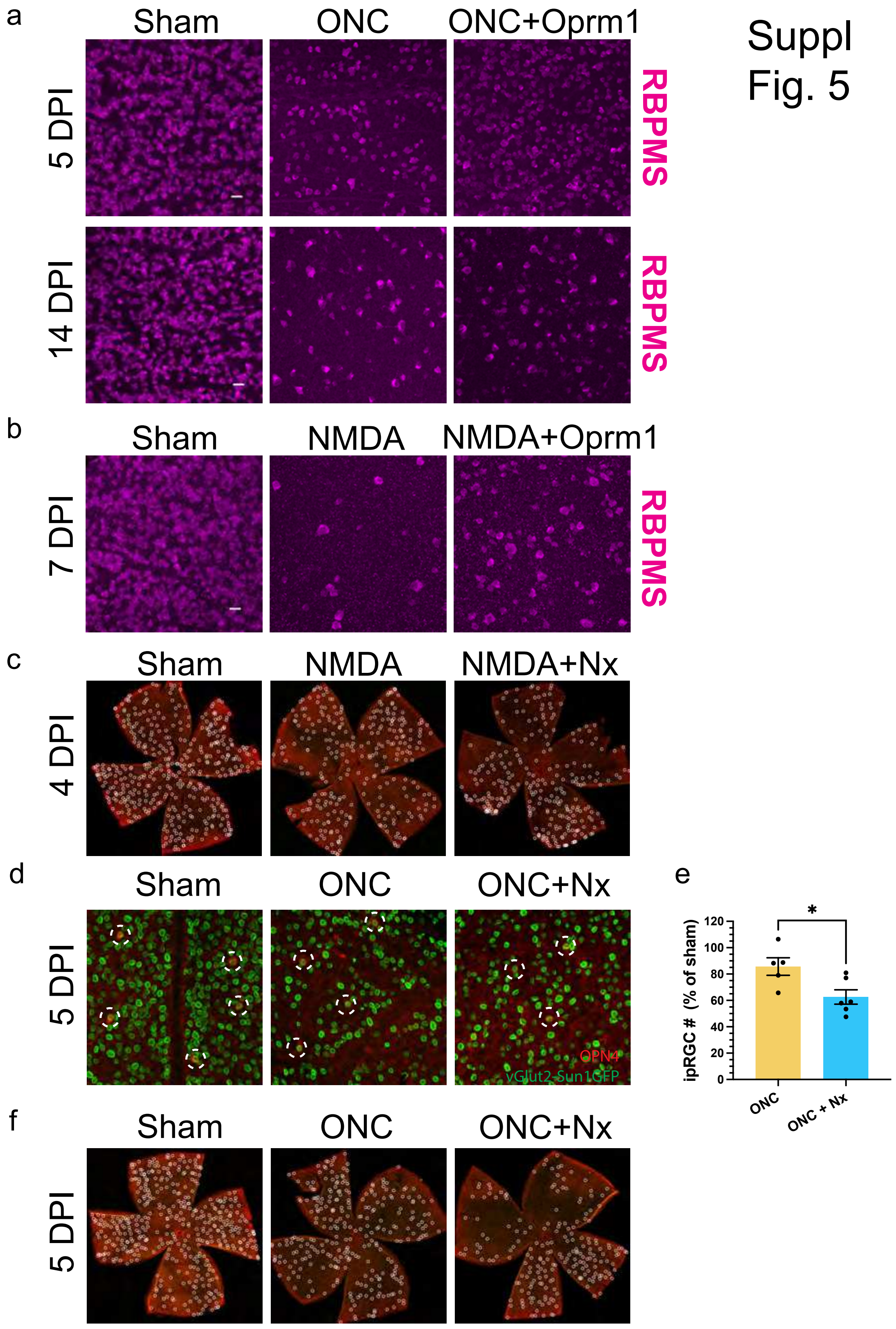

a
Sham
ONC
ONC+Oprm1
5 DPI
14 DPI
RBPMS
RBPMS
Suppl
Fig. 5
b
Sham
NMDA
NMDA+Oprm1
7 DPI
RBPMS
c
Sham
NMDA
NMDA+Nx
4 DPI
d
Sham
ONC
ONC+Nx
5 DPI
OPN4
vGlut2-Sun1GFP
e
*
ipRGC # (% of sham)
120
100
80
60
40
20
0
ONC
ONC + Nx
f
Sham
ONC
ONC+Nx
5 DPI

Suppl Fig. 6

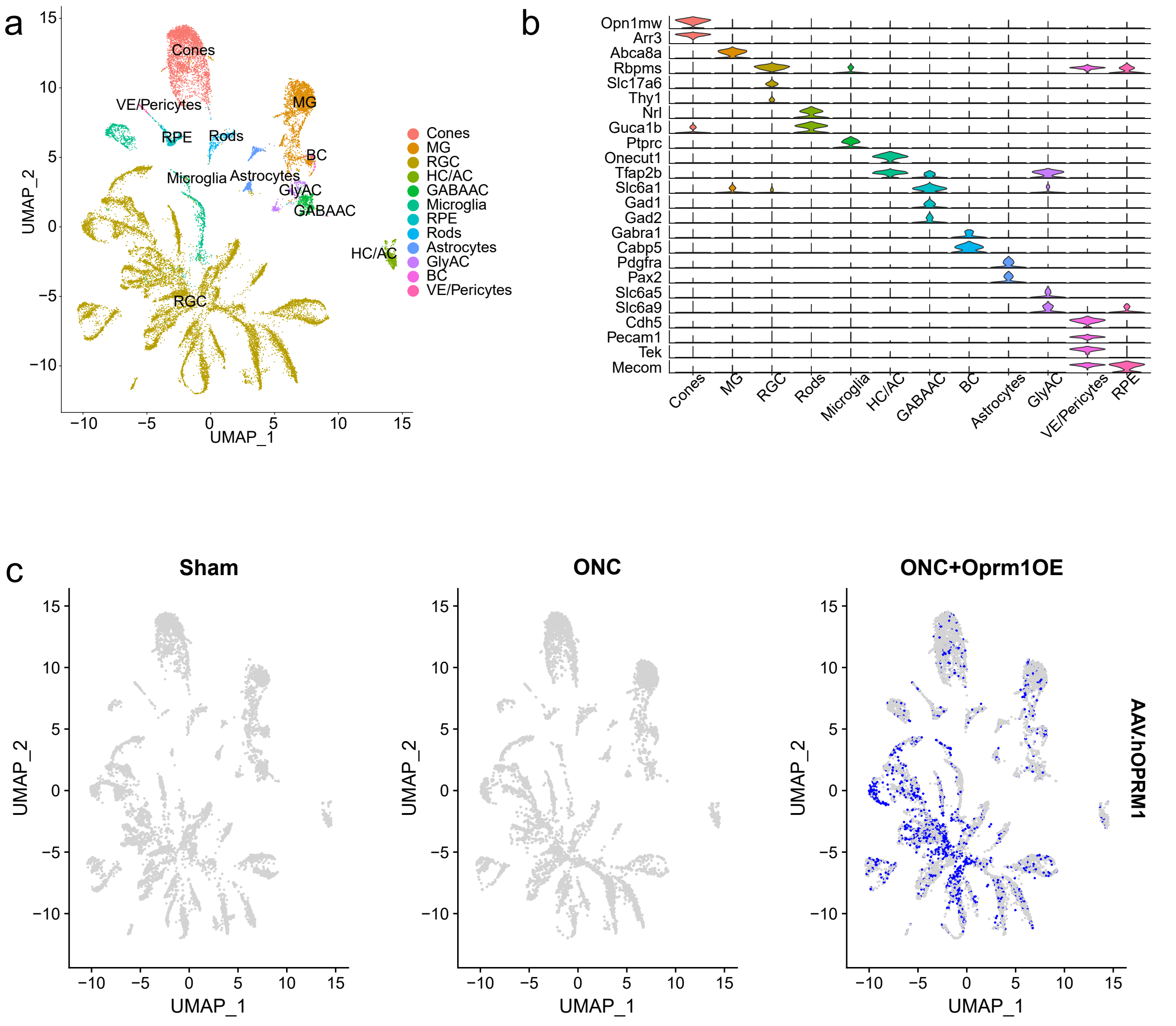
a
Cones
VE/Pericytes
MG
RPE
Rods
BC
Microglia
Astrocytes
GlyAC
GABAAC
HC/AC
RGC
UMAP_1
UMAP_2
Cones
MG
RGC
HC/AC
GABAAC
Microglia
RPE
Rods
Astrocytes
GlyAC
BC
VE/Pericytes
b
Opn1mw
Arr3
Abca8a
Rbpms
Slc17a6
Thy1
Nrl
Guca1b
Ptprc
Onecut1
Tfap2b
Slc6a1
Gad1
Gad2
Gabra1
Cabp5
Pdgfra
Pax2
Slc6a5
Slc6a9
Cdh5
Pecam1
Tek
Mecom
Cones
MG
RGC
Rods
Microglia
HC/AC
GABAAC
BC
Astrocytes
GlyAC
VE/Pericytes
RPE
c
Sham
ONC
ONC+Oprm1OE
UMAP_1
UMAP_2
AAV.hOPRM1

Suppl Fig. 7

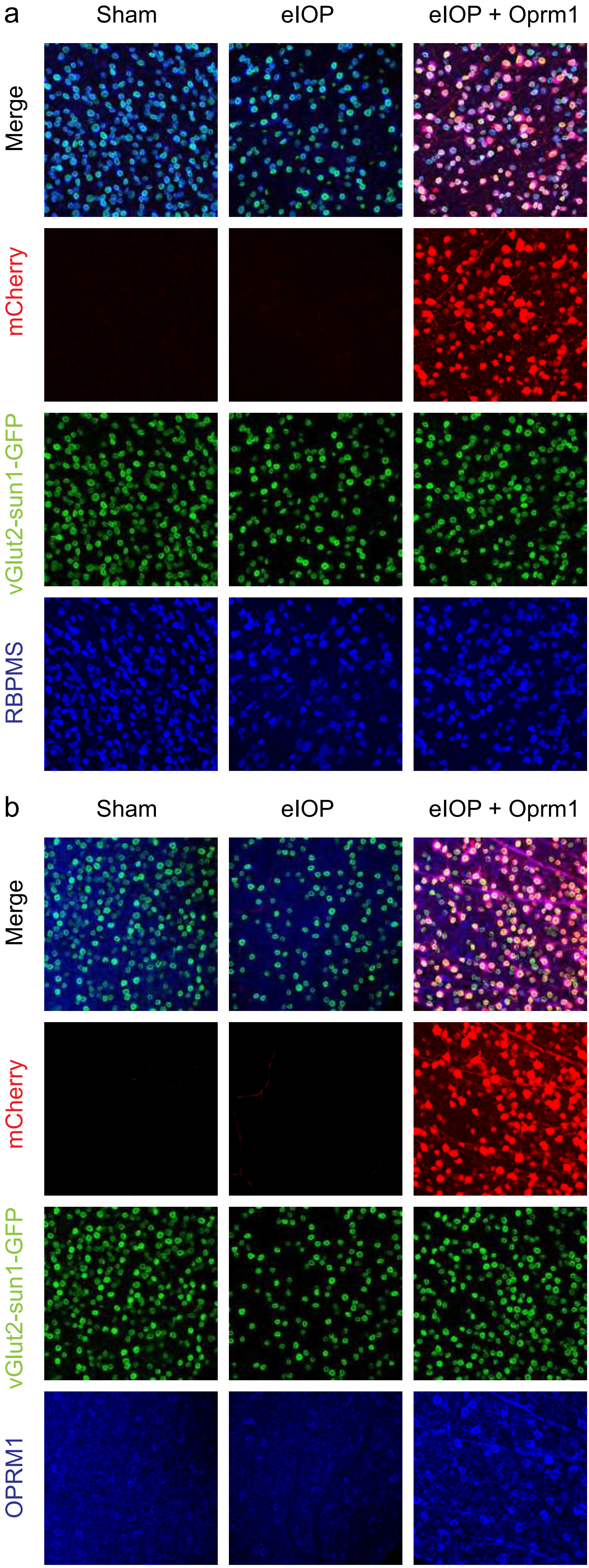
a
Sham
eIOP
eIOP + Oprm1
Merge
mCherry
vGlut2-sun1-GFP
RBPMS
b
Sham
eIOP
eIOP + Oprm1
Merge
mCherry
vGlut2-sun1-GFP
OPRM1
